# Clinical Applications and Mechanical Properties of CAD-CAM Materials in Restorative and Prosthetic Dentistry: A Systematic Review

**DOI:** 10.3390/jfb14080431

**Published:** 2023-08-17

**Authors:** Imena Rexhepi, Manlio Santilli, Gianmaria D’Addazio, Giuseppe Tafuri, Eugenio Manciocchi, Sergio Caputi, Bruna Sinjari

**Affiliations:** 1 Unit of Prosthodontics, Department of Innovative Technologies in Medicine and Dentistry, University “G. d’Annunzio” Chieti-Pescara, 66100 Chieti, Italy; imena.rexhepi@unich.it (I.R.); santilliman@gmail.com (M.S.); gianmariad@gmail.com (G.D.); giuseppe.tafuri@unich.it (G.T.); eugenio.manciocchi@unich.it (E.M.); scaputi@unich.it (S.C.); 2 Electron Microscopy Laboratory, University “G. d’Annunzio” Chieti-Pescara, 66100 Chieti, Italy

**Keywords:** CAD-CAM materials, digital dentistry, prosthodontics

## Abstract

Clinical outcomes of dental restorations depend primarily on the choice of materials used, and nowadays, dental CAD-CAM (Computer-Aided Design Computer-Aided Manufacturing) materials have strongly changed daily clinical practice. The aim of this systematic review is to analyze CAD-CAM dental materials according to their mechanical properties and in relation to their clinical applications. A literature review was performed on PubMed, Scopus, Web of Knowledge, and the Cochrane Library. Articles addressing at least one of the following topics regarding dental materials for CAD-CAM systems: manufacturers, mechanical features, materials’ composition, optical properties, clinical indications, and/or outcomes were included in the review. A flowchart was performed as described in the PRISMA guidelines. Among the 564 articles found, 63 were analyzed and evaluated. Within the limitations of this systematic review, it can be concluded that CAD-CAM materials present a wide range of clinical applications due to their improved mechanical properties. Specifically, in addition to materials that have been in use for a long time (such as feldspathic ceramics), resin block composites can also be used for permanent restorations.

## 1. Introduction

The introduction of “digital workflow” can be considered a turning point in dentistry [1]. The development of digital dentistry has led to an impressive change in daily clinical practice due to the synergy between new digital systems and considerable improvements in the mechanical and aesthetic features of dental materials. This reduces treatment times while maintaining high standards of precision and aesthetics [2,3]. Briefly, the dental digital workflow steps of CAD/CAM (Computer-Aided Design Computer-Aided Manufacturing) system are [4]:

(1) Scanning dental records by an intraoral scanner connected to dedicated software;

(2) Processing the digital data with a program that allows to visually design dental restorations;

(3) Manufacturing processes performed by subtractive (by milling it from a prefabricated block) or additive techniques [1].

**Figure 1:**
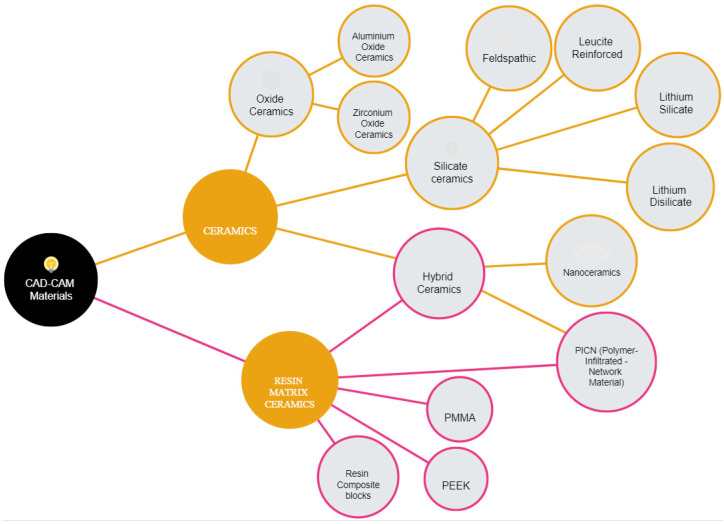
Classification of CAD-CAD materials based on their composition in dentistry.

CAD/CAM technology was first developed in the 1980s. The idea of this system was the result of the collaboration of three research centers, the University of Zurich with the Brains and Brandestini Instruments of Switzerland, Hennson International of France, and the University of Minnesota Center [5,6]. The authors’ purpose was to rehabilitate the patient with a prosthetic restoration in a short time and without the traditional impression-making method. The entire dental CAD-CAM system, including scanners, printers, and latest-generation software, is revolutionizing the manufacturing process [5,6,7]. Among its strengths, previous studies reported greater efficiency and comfort of digital scanning compared to conventional impressions [7,8]. In fact, CAD-CAM restorations offer a good combination of esthetics, durability, and functionality in a single restoration [9]. It was reported that CAD-CAM restorations, such as fixed dental prostheses supported by natural teeth and implants, have sufficient marginal adaptation and lead to reduced plaque accumulation with a lower incidence of periodontal inflammation and the development of caries [9,10]. Incorporation of an intraoral scanner reduces the procedure time, increases patient comfort, and allows to reach an adequate level of precision (4 to 80 microns for scans with a limited area) [10]. Nevertheless, CAD-CAM technology is still considered quite expensive, and its application requires highly trained personnel, with a learning curve that can range from a few days to several months [11]. In addition, the survival rate of CAD-CAM restorations may differ based on the types of materials used. It is impossible to date to make comparisons between the conventionally and digitally realized prosthetic restorations in terms of survival rate [10,11]. Several classifications of CAD-CAM materials have been described in the literature. One classification is made by materials, processing route, and type of manufacturing [12,13,14]. Among the classifications made by material, the CAD-CAM materials can be classified as silicate ceramics, oxide ceramics, composite resins, PMMA (polymethyl methacrylate), PEEK, and PICN polymer-infiltrated ceramic network materials, and of course metal [12]. Another type of classification is by its processing route; in fact, they can be classified as laboratory sides and chairside [13]. An additional classification recently considered is additive or subtractive manufacturing [14]. The spectrum of dental CAD-CAM materials covers a wide range of compositions [15], as shown in Figure 1. Each material has different processing parameters, and the whole system should be adapted based on the features of specific materials [16]. Dental CAD-CAM materials differ according to their composition, and their mechanical and physical properties guide dental practitioners in selecting the most appropriate material to be used [15,16]. The aim of this systematic review is to explore the clinical applications of these materials and their correlation with the enhancement of their mechanical characteristics.

## 2. Materials and Methods

### 2.1. Search Strategy 

**Figure 2:**
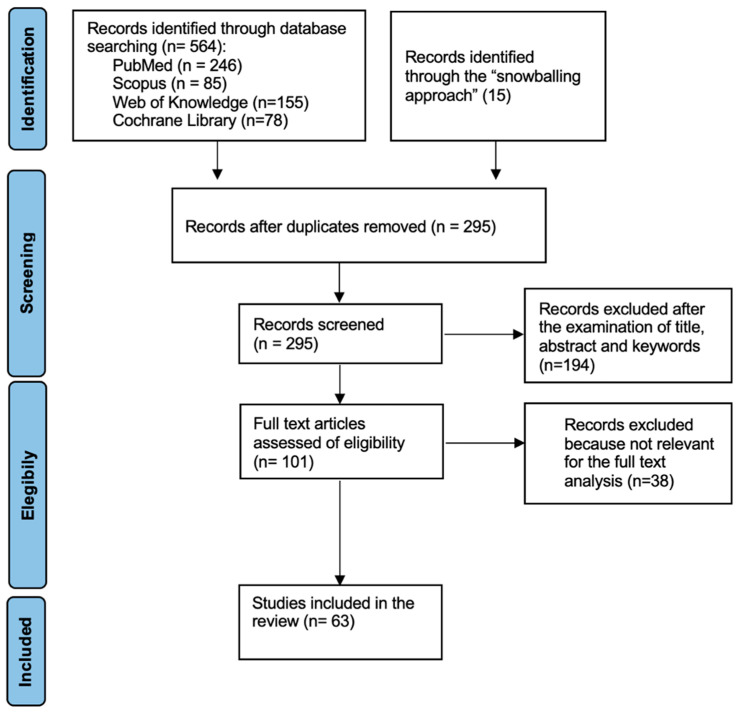
The flow diagram reports the decisions the review team took as they assessed citations for possible inclusion in the review. The search flowchart is described in the PRISMA guidelines. Caption: (n = number of records).

This systematic review adhered to the PRISMA (Preferred Reporting Items for Systematic Reviews and Meta-analyses) guidelines and employed the PICO(S) framework (Patient or Population, Intervention, Control or Comparison, Outcome, and Study types) [17], as illustrated in Figure 2. Thorough research into the literature and papers concerning dental materials used in CAD-CAM restorations was conducted across databases, including PubMed (Medline), Scopus, Web of Knowledge, and the Cochrane Library. Furthermore, the “snowballing” method was applied to uncover additional papers by scrutinizing the reference lists of already-identified records [18].

The research process encompassed employing combinations of the subsequent keywords: “computer-aided design” OR “CAD/CAM” OR “digital dentistry” AND “dental materials” OR “prosthetic dentistry” OR “restorative dentistry”. The scope of the search was limited to English language articles, and the electronic scan encompassed papers published within the last five years. The cutoff point was set in 2017, considering the considerable advancements in scanner hardware and software, as well as dental CAD-CAM material science, that transpired from January 2017 to January 2022 [14]. A hybrid approach of free-text keywords was employed in the search strategy. To eliminate duplicates, references from the identified records were integrated as Research Information Systems files into Zotero (RRCHNM, Fairfax, VA, USA). The search strategy, aligned with the focused PICOS question, is detailed in Table 1.

### 2.2. Inclusion and Exclusion Criteria

Articles were considered appropriate when satisfying the following inclusion criteria (as shown in Table 2):

(1) Articles addressing at least one of the following topics regarding dental materials for CAD-CAM systems: clinical indications and/or outcomes, manufacturers, mechanical features (flexural strength, hardness, and elastic modulus), and materials’ composition or optical properties;
(2) Studies performed in vitro or in vivo;
(3) Systematic and narrative reviews.

Articles that did not have the above information were excluded from the review.

## 3. Results

The search strategy reported 564 records, including duplicates: 246 from PubMed, 85 from Scopus, 155 from Web of Knowledge, 78 from the Cochrane Library, and 15 with the “snowballing” approach. The duplicates were eliminated; thus, all the selected databases produced 295 records. After the analysis of titles, abstracts, and mesh words, the researchers excluded 194 records that did not meet the inclusion criteria. Among the remaining 101 studies, 38 more were excluded after a full-text examination since these records did not present considerable information regarding the clinical applications of CAD/CAM dental materials in daily practice. The remaining 63 articles were included in this systematic review. The workflow of the review screening process is reported in Figure 2, in accordance with the “PRISMA 2009 Flow Diagram” [17,18]. Data obtained are the result of the correlation of the chemical–physical properties of the CAD-CAM materials in relation to their clinical outcomes, as reported in Table 3 and Table 4.

## 4. Discussion

### 4.1. Silicate Ceramics (Glass Ceramics)

Chemically silica-based ceramics are non-metallic materials containing a glassy matrix. Generally, the inclusion of glass within their compositions contributes to traits such as brittleness, reduced fracture strength, and resistance [19]. These ceramics possess translucency, exhibiting exceptional optical characteristics that stem from their inherent natural appearance. Moreover, they necessitate hydrofluoric (HF) acid etching, a step aimed at augmenting micromechanical adhesion, and adhesive bonding, which serves to enhance mechanical properties. Upon acid etching, the glassy matrix dissolves, unveiling a crystalline phase that renders the ceramic surface suitable for resin cement interlocking. Within the realm of silicate ceramics, noteworthy types encompass traditional feldspathic ceramics, lithium silicate, lithium disilicate, and leucite-reinforced ceramics [20].

#### 4.1.1. Feldespathic 

This type of silicate ceramic is the first one used with CAD-CAM systems, especially by chairside. From a chemical point of view, they are considered ternary material systems composed of clay/kaolin, quartz (silica), and naturally occurring feldspar (a mixture of potassium and sodium aluminosilicate). Potassium feldspar (K_2_A_12_Si_6_O_16_) forms leucite crystals (crystalline phase) which, depending on the amount, increase the intrinsic strength of the restoration [21]. 

They have excellent optical properties; in fact, their color and translucency are close to natural teeth, even if among the glass-based ceramics, they are the weakest ones as they tend to fracture. In addition, they require etching with 9.6% hydrofluoric acid for 1 min and then the silane application. That is why they are indicated for inlay, onlay, anterior and posterior restorations, and, in general, for veneers (also for veneering metal substructures, with a coefficient of thermal expansion of approximately 10% or less) [21]. Cerec blocs Dentsply Sirona, Pennsylvania, and Vitabloc Mark II, Real-Life, TriLuxe, and VITA Zahnfabrik are the most representative blocs of this category [21].

#### 4.1.2. Leucite-Reinforced 

They are particle-filled glasses with a composition of the synthetic category (leucite-based, up to 45%) depending on the manufacturers [21,22]. In the literature, several benefits have been reached by reinforcing the matrix with leucite, thus improving flexural strength (up to 104 Mpa) [22]. In addition, the reinforcement through leucite allows it to have a high thermal contraction coefficient [22]. Moreover, leucite-based ceramics present good translucency properties because their index of refraction is close to feldspathic glasses. Leucite reinforcement also permits a selected etching, thus ameliorating the micromechanical bond [16]. Leucite-reinforced materials have high translucency and excellent optical properties, making it preferable to use them in esthetic areas compared to non-load-bearing areas. A commercial example is represented by IPS Empress CAD, IPS Classic, and Ivoclar Vivadent [20]. They are indicated for veneers, inlays, onlays, and single crowns [20]. Moreover, etching with 5% HF for 20 s is recommended for leucite-reinforced CAD/CAM ceramics [23,24].

#### 4.1.3. Lithium Silicate 

Also, lithium silicate ceramics are included among silicate ceramics and could be considered a progression of silicate ceramics [25]. From a chemical point of view, they are composed of a crystalline phase (lithium disilicate) and lithium orthophosphate. The mechanical strength of lithium silicate ceramics is increased thanks to the homogeneously dispersed crystalline phase. It is frequently enriched with zirconia (approximately 10% of zirconia dioxide), thus combining very high mechanical properties and optical characteristics [26]. Lithium silicates have a flexural strength of around 400 MPa and good color stability compared to high-translucency zirconia or nanoceramics. VITA Suprinity PC by VITA Zahnfabrik and Celtra Duo by Dentsply Sirona are examples of lithium silicate ceramics. The use of silicate ceramics is limited to single crowns (better in anterior regions), veneers, inlays/onlays, and leucite-reinforced CAD/CAM ceramics [23,24].

#### 4.1.4. Lithium Disilicate 

Lithium disilicate (Li_2_Si_2_O_5_) is characterized by the presence of approximately 65% lithium disilicate crystals, measuring between 2 and 5 μm in length and 0.8 μm in diameter, embedded within an amorphous glassy matrix, classifying it as a glass-ceramic material. The chemical composition (Li_2_Si_2_O_5_) contributes to lithium disilicate’s remarkable mechanical attributes, including a flexural strength of 350 MPa, fracture toughness (KIC) of 3.3 MPa m^½^, heat extrusion temperature of 920 °C, thermal expansion coefficient (CTE) of 10.6 + 0.25 ppm/°C, and notably high translucency [27,28,29].

The exceptional translucency of these ceramics renders them a favored choice for aesthetically demanding cases, albeit the variability in flexural strength of CAD-CAM blocks hinges on the manufacturer [30]. These ceramic blocks have demonstrated noteworthy clinical success when employed in non-load-bearing regions, while their robust mechanical characteristics allow for expanded applications, particularly in veneers, inlays/onlays, single crowns, or small bridges (up to 3 units). IPS e.max CAD from Ivoclar Vivadent stands as a prominent example within this category of materials, boasting a flexural strength of approximately 360 MPa. Commercial instances also include VITA Suprinity PC from VITA Zahnfabrik, Celtra Duo from Dentsply Sirona, and Obsidian from Glidewell Laboratories in Newport Beach, California [27,30]. Furthermore, the recommended practice for lithium disilicate CAD/CAM ceramics involves etching with 5% hydrofluoric acid (HF) for a duration of 20 s [23,24].

### 4.2. Oxide Ceramics

These materials have very favorable mechanical properties, and they are mainly suggested for crowns, implant components, and Fixed Dental Prostheses (FDPs) with multiple units in anterior and posterior areas, even if their aesthetic properties are somewhat inferior to silicate ceramics [26]. Oxide ceramics refer to inorganic compounds composed of metallic or metalloid elements, including aluminum (Al), zirconium (Zr), titanium (Ti), magnesium (Mg), and silicon (Si), combined with oxygen (O). These materials possess outstanding mechanical properties, resistance to corrosion, and durability, rendering them suitable for a wide range of applications [31]. Since oxides represent the highest oxidation state of metals, they exhibit remarkable stability even in the most challenging industrial processes and application conditions. Research has strongly demonstrated the biocompatibility of oxide-based ceramics [31,32]. Furthermore, porous ceramic structures have been utilized as a method to facilitate bone regrowth and mechanically interlock prostheses [32]. During the late 1970s, alumina (Al_2_O_3_) gained significant attention as a ceramic biomaterial due to its robustness and compatibility with living tissues. Later, zirconium dioxide emerged as an alternative to Al_2_O_3_, offering relatively high fracture strength as another promising option in the field of ceramic biomaterials [32].

#### 4.2.1. Zirconium Oxide Ceramics 

Zirconia, from a chemical standpoint, represents a metal oxide endowed with polymorphism and allotropy attributes, positioning it as an “all-ceramic” material within the realm of dentistry. Furthermore, it displays distinct crystallographic structures, including monoclinic, tetragonal, and cubic, which contribute to its diverse mechanical and optical traits [27]. At temperatures surpassing 2370 °C, zirconium oxide adopts a cubic structure, transitioning to a tetragonal structure between 2370 °C and 1170 °C and a monoclinic structure below 1170 °C. Consequently, upon cooling to room temperature, zirconia assumes a monoclinic configuration, which, unfortunately, exhibits limited resistance to cyclic mechanical stress. These ceramics are formulated in the form of Yttria-stabilized tetragonal zirconia polycrystal (Y-TZP). Incorporating yttrium oxide in varying concentrations (3–5%) leads to a reduction in their mechanical attributes. Notably, Y-TZP showcases optimal mechanical traits, boasting a remarkable fracture resistance ranging from 5 to 10 MPa m^½^, alongside a flexural strength spanning 900–1400 MPa [28]. Commercial exemplars encompass Nobelprocera Zirconia from Nobel Biocare and Lava Plus from 3M ESPE. A number of articles have evidenced a survival rate of up to 100% for fixed dental prostheses (FDPs) rehabilitation after a span of 5 years [33,34].

Owing to their exceptional mechanical features, particularly their optical properties, the utility of these CAD-CAM materials spans a broad spectrum, extending to applications such as single crowns or comprehensive rehabilitations involving multiple units (bridges in both anterior and posterior regions, encompassing full-arch rehabilitations involving implants or natural teeth) [35].

#### 4.2.2. Aluminum Oxide Ceramics

These ceramics consist of a core composition of glass-infiltrated aluminum oxide. Precisely, the chemical makeup comprises densely compacted sintered Al_2_O_3_ (comprising 80 to 82 wt%) as the core ceramic material, which is subsequently subjected to infiltration with molten glass. With a flexural strength reaching approximately 500 MPa, these ceramics come highly recommended for crafting anterior three-unit fixed dental prostheses, as well as crowns, making them a suitable choice for posterior restorations [36]. Among the most renowned commercial instances is InCeram Alumina (VITA Zahnfabrik, Bad Sackingen, Germany) [36].

In a broader context, alumina crowns exhibit commendable long-term survival rates, even boasting up to 100% survival rates over a span of 7 years [37,38,39].

### 4.3. Hybrid Ceramics 

#### 4.3.1. PICN

Polymer-infiltrated ceramic network (PICN) material has both ceramic and polymer properties. It is defined as a double mesh hybrid material with ceramic and polymer. The PICN polymerization process involves the production of a pre-sintered porous ceramic network that is infiltrated by a polymer in a capillary manner. PICN has better wear resistance than composite resins and exhibits high flexural strength and elasticity like that of dentin [40]. The dominant ceramic network demonstrates good wear resistance, and the interpenetration of ceramic and polymer prevents crack propagation in the material. As a newly introduced material, there are still no studies with long-term follow-up PICN restorations. The color range of the material is limited; there are no adequate follow-ups for its durability on cervical areas and discolorations [41]. The PICN is indicated for veneers, inlays/onlays, anterior and posterior single crowns, and implant prostheses. However, this material is more suitable for posterior reconstructions due to the lower aesthetic yield [40,41]. PICN composite CAD/CAM blocks have found application in the realm of indirect tooth restoration, with numerous foundational and clinical investigations employing a commercially accessible PICN composite referred to as VITA ENAMIC. This composite is characterized by a silicate glass ceramic framework infused with acrylic resin [42]. Earlier research has highlighted the ability of PICN composites, exemplified by VITA ENAMIC, to effectively replicate the mechanical characteristics of human enamel [43,44].

#### 4.3.2. Nanoceramics

Nanoceramics exhibit a comparable microstructure to resin composites but with distinct proportions. Comprising a polymeric matrix and ceramic nanoparticles as fillers (each less than 100 nm in size), they typically constitute around 80% of the total weight. These nanoparticles can consist of conventional ceramics, polycrystalline ceramics (such as zirconia), or a hybrid blend of the two [45]. This sets nanoceramics apart from resin composite blocks primarily in terms of filler-to-polymer ratio and particle size. Nanoceramics bear similarities to natural teeth, often featuring a flexural strength nearing 200 MPa, compressive strength reaching up to 380 MPa, and an abrasion rate averaging between 2 and 10 microns annually. Their elastic modulus hovers around 15 GPa [46,47]. Such attributes render nanoceramics suitable for single tooth restoration or minor bridges, ideally positioned in posterior regions and conceivably applicable in anterior cosmetic treatments [47]. Nonetheless, it is worth noting that the polymer matrix in nanoceramics is more prone to wear than the ceramic component, making them comparatively more abrasive to opposing teeth compared to traditional ceramics [48]. Nanoceramics find an indication in various dental procedures, including veneers, inlay/onlay applications, and both anterior and posterior single crowns and bridges [47,48].

Lava Ultimate (3M ESPE) is a prominent example within the nanoceramics category, specifically designed for compatibility with CAD/CAM systems [49]. The strong chemical bonds established between the nanoceramic structure and resin contribute to its impressive fracture strength. Moreover, Lava Ultimate exhibits a flexural strength of 200 MPa [50]. Notably, the elastic modulus of Lava Ultimate, reported by Lauvahutanon et al., is approximately 29.8 GPa, remarkably akin to dentin, which implies a substantial capacity to absorb forces. This characteristic lends itself to the fabrication of posterior nanoceramic restorations [51,52].

### 4.4. Resin Matrix Ceramics

#### 4.4.1. PMMA

PMMA (IUPAC name: poly [1-(methoxy carbonyl)-1-methyl ethylene]) emerges as a synthetic polymer synthesized through the free radical addition and polymerization of methyl methacrylate (C_5_O_2_H_8_) to form poly methyl methacrylate (C_5_O_2_H_8_)_n_ [53]. Within the realm of dentistry, PMMA has garnered favor due to its distinctive attributes, including its diminished density, pleasing aesthetics, cost-effectiveness, ease of manipulation, and versatile physical and mechanical characteristics [54].

The burgeoning interest in PMMA restorations spurred the evolution of PMMA blocks characterized by enhanced optical and physical properties. Examples include Telio CAD from Ivoclar Vivadent, Shlan from Liechtenstein, and VITA CAD-Temp MultiColor Blocks from VITA Zahnfabrik (Bad Sackingen, Germany) [55,56,57]. Heat-cured PMMA restorations are amenable to polished finishes, elevating their aesthetic appeal. PMMA finds wide application in prosthodontic dental contexts, encompassing the crafting of synthetic teeth, denture bases, complete dentures, obturators, orthodontic retainers, provisional or temporary crowns, as well as dental prosthesis repairs [57].

Despite the shared chemistry with conventionally heat-cured PMMA, CAD/CAM PMMA exhibits superiority in terms of hardness, flexural strength, flexural modulus, and impact resistance [58]. The enhancement of these mechanical attributes has extended the usage of CAD/CAM PMMA even for long-term provisional restorations (up to one year) [59,60,61]. Furthermore, the augmented hydrophobic nature of CAD/CAM PMMA, in contrast to its conventional counterpart, results in reduced plaque accumulation on the surfaces of CAD/CAM prostheses. Notably, diminished adhesion of Candida albicans, the primary pathogen in prosthetic stomatitis, has also been documented [62].

#### 4.4.2. PEEK

Polyetheretherketone (PEEK), a semi-crystalline thermoplastic polymer, finds application within the dental domain as a versatile choice for metal-free frameworks. Its utility extends to removable fixed dental prostheses, fixed prostheses upheld by implants, overdentures anchored by implants, endo-crowns, and resin-bonded fixed dental prostheses [63]. PEEK showcases commendable wear resistance, a diminished propensity for plaque retention, and robust adhesive capabilities with veneering composites and luting cement. Furthermore, it possesses a modest modulus of elasticity at 4 GPa, akin to the elasticity of bone. This characteristic imparts a cushioning effect, leading to a reduction in the transfer of stresses to the abutment teeth [64].

Comparative assessments have been conducted between conventional techniques and CAD/CAM-fabricated PEEK dentures, revealing comparable or, in certain instances, superior fit with the latter approach [65,66]. PEEK has demonstrated a more favorable outcome in two-body wear tests when pitted against other CAD/CAM composite resin and PMMA materials. In vitro trials simulating chewing stresses evaluated PEEK molar crowns constructed on zirconia and titanium abutments, yielding satisfactory fracture strength properties and endorsing their suitability for clinical application [67].

Nonetheless, despite these promising attributes, PEEK currently remains unavailable for clinical use due to the dearth of comprehensive clinical studies attesting to its performance [68].

Moreover, PEEK exhibits remarkable abrasive properties [68]. Despite having notably lower elastic moduli and hardness, its abrasive resistance rivals metallic alloys [66,67,68]. Nonetheless, there have been no clinical endeavors to directly contrast the abrasion caused by PEEK crowns on teeth with that induced by other materials like alloys and ceramics [63,68]. Consequently, it remains uncertain whether PEEK crowns can effectively coexist with dentin and enamel. Given its favorable abrasion resistance, mechanical characteristics, and the previously mentioned strong bond to composites and teeth, a PEEK fixed partial denture is anticipated to exhibit a satisfactory rate of survival [68].

Among the most used CAD/CAM PEEK manufacturers, PEEK “blanks” (Juvora dental PEEK CAD/CAM-Rohling, Straumann, Basel, Switzerland) can be applied to mill frameworks for dentures or FDPs and BioHPP™ (Bio High-Performance Polymer, Bredent, Senden, Germany) is approved by the manufacturer for three to four-unit FDPs, telescopic restorations, implant abutments, and secondary structures associated with bar-supported prostheses [69,70].

#### 4.4.3. Resin Composite Blocks (RCBs)

Resin composite blocks (RCBs) designed for CAD/CAM are produced by the incorporation of filler particles into a mixture of monomers that are cured under high temperature and high pressure [71,72,73]. This polymerization method allows, compared to traditional resin composites, a greater homogeneity of the material with a lower presence of defects and pores and a greater degree of conversion that reduces the absorption of water, improving mechanical features such as resistance to fracture, resistance to bending and wear [74]. Most CAD/CAM RCBs have urethanodimethacrylate (UDMA) as a polymer matrix with lower solubility and water absorption capacity, allowing the restorations to have greater color stability [75]. Furthermore, these new dimethacrylates have an addition-fragmentation monomer with an enhancement of their translucency, which can promote the increase of DC and hardness (VH) during polishing procedures, achieving an optimum clinical performance [76]. The Paradigm MZ100 (3M Oral Care, Seefeld, Germany) was the first CAD/CAM composite material with a flexural strength of 157 MPa, similar to feldspar ceramic materials [77]. Other examples of RCB CAD/CAM include:

Tetric CAD (Ivoclar Vivadent, Liechtenstein) is a resinous matrix consisting of Bis-GMA, Bis-EMA, TEGDMA, and UDMA, filled with 70% barium glass and silicon dioxide particles. This composite has a flexural strength of 273.8 MPa and an elastic modulus of 10.2 GPa [12].

LuxaCam Composite (LUXA) (DMG; Hamburg, Germany) is a resin matrix composed of 70% silicate-glass filling particles. This composite demonstrates a flexural strength of 164 MPa and an elastic modulus of 10.1 GPa. [78].

Grandio Blocks (VOCO GmbH, Cuxhaven, Germany) is a resin matrix highly nanohybrid filled (86%) with a flexural strength of 330 MPa and an elastic modulus of 18 GPa, offer physical properties that mimic natural human tissues, such as thermocycling [79,80].

In addition, RBCs for CAD/CAM procedures exhibit higher color stability than direct or indirect (laboratory) RBCs and lower color stability than the ceramic materials; In fact, the color stability of these materials results from the material composition and by finishing/polishing techniques have an impact [81].

These CAD/CAM composites, according to the manufacturers’ indications, can be used to perform inlays, onlays, veneers, partial crowns, crowns, and multi-unit, up to three bridge units due to the two levels of translucencies (HT, LT) to better reproduce natural teeth optical characteristics [82].

## 5. Conclusions

Within the limitations of this systematic review, it can be deduced that silicate ceramics demonstrate a high success rate for single-tooth restoration. For anterior restorations, lithium silicate and disilicate are recommended due to their excellent translucency. Zirconium is utilized for bridges in both the anterior and posterior regions, and it is even employed in full-arch rehabilitations involving implants or natural teeth. Hybrid ceramics, on the other hand, find utility in inlays/onlays and posterior single crowns, with PICN (polymer infiltrated ceramic network) being favored due to its greater strength over aesthetics. Among Resin Matrix Ceramics, PMMA (polymethylmethacrylate) serves as a suitable choice for temporary or provisional crowns, while PEEK (polyetheretherketone) proves to be a valuable option for metal-free structures, endocrowns, and fixed dental prostheses. In recent times, resin composite blocks have gained popularity as they have undergone improvements in mechanical and aesthetic properties. As a result, they are now considered viable alternatives for definitive restorations, such as inlays, onlays, veneers, partial crowns, and bridges, including multi-unit bridges with up to three units.

## Figures and Tables

**Table 1:** Search strategy according to the focused question (PICO).

Focused Question (PICO)	Is There a Greater Range of Clinical Applications of CAD/CAM Materials than Traditional Ones Due to the Improvement of Their Mechanical Properties?	
Search strategy	Population	Teeth to be partially or totally rehabilitated
	Intervention	CAD/CAM restorations teeth to be partially or totally rehabilitated
	Comparison	CAD/CAM restorations teeth to be partially or totally rehabilitated compared to Conventionally manufactured restorations due to their mechanical properties
	Outcome	Clinical Application of these materials in accordance with their mechanical properties

**Table 2:** Inclusion Criteria of the Systematic Review.

Inclusion Criteria
Articles addressing at least one of the following topics regarding dental materials for CAD-CAM systems
clinical indications and/or outcomes; manufacturers; mechanical features (flexural strength, hardness, and elastic modulus); materials’ composition; optical properties.
In Vivo Studies
In Vitro Studies
Systematic Reviews
Narrative Reviews

**Table 3:** Clinical application of CAD/CAM materials included in the study.

Materials	Clinical Application	References
Silicate Ceramics		
Feldespathic	inlay, onlay, anterior and posterior restorations and for veneers	Skorulska, A. et al. (2021) [19], Zhang Y. et al. (2018) [20], Gracis, Stefano et al. (2015) [21]
Leucite-reinforced	veneers, inlays, onlays, and single crowns	Gracis, Stefano et al. (2015) [21], H Ahmed et al. (2019) [22], Avram et al. (2022) [23], Veríssimo et al. 2019 [24]
Lithium silicate	single crowns (better in anterior regions), veneers and inlays/onlays	Hinz, Sebastian et al. (2022) [25] D’Addazio, Gianmaria et al. (2020) [26]
Lithium disilicate	veneers, inlays/onlays, single crowns or small bridges (up to 3 units)	Hinz, Sebastian et al. (2022) [25] D’Addazio, Gianmaria et al. (2020) [26] Mavriqi, Luan et al. (2021) [27] Fabian Fonzar et al. (2017) [28] Gardell E. et al., (2021) [29] Traini, Tonino et al. (2014) [30]
**Oxide Ceramics**		
Zirconium	bridges in anterior or posterior region, up to entire full-arch rehabilitations on implants or natural teeth	Mirdamadi E.S. et al. (2021) [31] Li J. et al. (1998) [32] Guazzato, Massimiliano et al. (2004) [33] Monaco, Carlo et al. (2015) [34] Pihlaja, Juha et al. (2016) [35] Joda, Tim et al. (2021) [36]
Aluminum	anterior three-unit fixed dental prosthesis, crowns and for posterior rehabilitation	llenz, Maximiliane Amelie et al. (2021) [37] Ozer, Fusun et al. (2014) [38] Selz, Christian F et al. (2014) [39]
**Hybrid Ceramics**		
Polymer infiltrated ceramic network (PICN)	veneers, inlays/onlays, anterior and posterior single crowns and for implant prostheses	Kawajiri, Yohei et al. (2021) [40] Kang, Longzhao et al. (2020) [41] Steinbrenner, Harald (2018) [42] Yano, Haruka Takesue et al. (2020) [43] Li, Ke et al. (2021) [44]
Nanoceramics	veneers, inlay/onlay, anterior and posterior single crowns, anterior and posterior bridges	Demirel, Akif et al. (2017) [45] Heck, Katrin et al. (2019) [46] Al Amri, Mohammad D et al. (2021) [47] Al-Harbi, Fahad A et al. (2017) [48] Yin, Ruizhi et al. (2019) [49] Ludovichetti, Francesco Saverio et al. (2018) [50] Lauvahutanon, Sasipin et al. (2017) [51] Kurtulmus-Yilmaz, Sevcan et al. (2019) [52]
**Resin Matrix Ceramics**		
Polymethyl methacrylate (PMMA)	long term (up to one year) provisional restoration	Zafar, Muhammad Sohail (2020) [53] Hassan, M et al. (2019) [54] Arslan, Mustafa et al. (2018) [55] Al-Dwairi, Ziad N et al. (2018) [56] Al-Dwairi, Ziad N et al. (2019) [57] Bidra, Avinash S et al. (2013) [58] Choi, Joanne Jung Eun et al. (2020) [59] Kalberer, Nicole et al. (2019) [60] de Oliveira Limírio, João Pedro Justino et al. (2021) [61] Murat, Sema et al. (2019) [62]
Polyether Ether Ketone (PEEK)	mill frameworks for dentures or FDPs, three to four-unit FDPs, telescopic restorations, implant abutments, and secondary structures associated with bar-supported prostheses	Papathanasiou, Ioannis et al. (2020) [63] Alexakou, E et al. (2019) [64] Muhsin, S.A et al. (2018) [65] Peng, Tzu-Yu et al. (2020) [66] Negm, Enas Elhamy et al. (2019) [67] Najeeb, S et al. (2016) [68] Wang, Jing et al. (2021) [69] Arnold, Christin et al. (2018) [70]
Resin Block Composites	inlays, onlays, veneers, partial crowns, crowns, and multi-unit, up to three bridge units	Alamoush, Rasha A et al. (2018) [71] Alamoush, Rasha A et al. (2022) [72] Fonseca, Andrea Soares Q S et al. (2017) [73] Marchesi, Giulio et al. (2021) [74] Liebermann, Anja et al. (2016) [75] Monterubbianesi, Riccardo et al. (2020) [76] Alharbi, Amal et al. (2017) [77] Schlenz, Maximiliane Amelie et al. (2019) [78] Vichi, Alessandro et al. (2020) [79] Wendler, Michael et al. (2021) [80] Paolone G. et al. (2023) [81] Vichi Alessandro et al. (2023) [82]

**Table 4:** Summary of the CAD/CAM Materials included in the study and related to their mechanical properties.

Mechanical Properties:	Flexural Strength (MPa)	Vickers Hardness (VH)	Elastic Modulus (GPa)	References	Manufacturers
Silicate Ceramics					
Feldespathic	97–133	640 ± 20	45	[19,20,21]	CEREC Blocs (VITABLOC, Bad Säckingen, Germany)
Leucite-reinforced	106–160	525–565	62–70	[21,22,23,24]	IPS Empress CAD, (Ivoclar Vivadent, Liechtenstein)
Lithium silicate	400	up to 7000	70	[25,26]	Suprinity PC (Vita Zahnfabrik, Bad Säckingen, Germany), Celtra Duo (Densply Sirona, Verona, Italy)
Lithium disilicate	130	452–731	58–110	[25,26,27,28,29,30]	IPS E. max CAD (Ivoclar Vivadent, Liechtenstein)
**Oxide Ceramics**					
Zirconium	500–1200	12	210	[31,32,33,34,35,36]	Nobelprocera Zirconia (Nobel Biocare, Kloten, Switzerland) Lava Plus, (3M ESPE, Milano, Italy)
Aluminum	500	18.3	206	[37,38,39]	InCeram Alumina (Vita Zahnfabrik, Bad Säckingen, Germany)
**Hybrid Ceramics**					
Polymer-infiltrated ceramic network (PICN)	107.8–153.7	204.8–299.2	13.0–2.2	[40,41,42,43,44]	VITA ENAMIC (Vita Zahnfabrik, Bad Säckingen, Germany),
Nanoceramics	200	91.5	15	[45,46,47,48,49,50,51,52]	Lava Ultimate (3M ESPE, Milano, Italy)
**Resin Matrix Ceramics**					
Polymethyl Methacrylate (PMMA)	80–135	27.7411	2.68–3.43	[53,54,55,56,57,58,59,60,61,62]	Telio CAD, Ivoclar Vivadent, VITA CAD-Temp MultiColor Blocks, (Vita Zahnfabrik, Bad Säckingen, Germany),
Polyether Ether Ketone (PEEK)	165–185	26.1–28.5	4	[63,64,65,66,67,68,69,70]	Juvora dental PEEK CAD/CAM-Rohling, Straumann, Bio High Performance Polymer, (Bredent, Senden, Germany)
Resin Block Composites	80	65–98	2.8	[71,72,73,74,75,76,77,78,79,80,81,82]	Grandio Blocks (VOCO GmbH, Cuxhaven, Germany), LuxaCam Composite (LUXA, DMG, Cheshire, UK)
